# Prognostic factors in patients with gastrointestinal perforation under the acute care surgery model : a retrospective cohort study

**DOI:** 10.1186/s12893-024-02687-7

**Published:** 2024-12-21

**Authors:** Kiyoung Sung, Sanguk Hwang, Jaeheon Lee, Jinbeom Cho

**Affiliations:** 1 Department of Surgery, Bucheon St. Mary’s Hospital, College of Medicine, The Catholic University of Korea, Seoul, Republic of Korea; 2 Department of Artificial Intelligence, The Catholic University of Korea, Bucheon, Republic of Korea

**Keywords:** Acute care surgery, Gastrointestinal perforation, Prognosis, Machine learning

## Abstract

**Background:**

Gastrointestinal perforation (GIP) is a life-threatening condition that necessitates immediate surgical intervention. This study aims to identify prognostic factors in patients with GIP treated within a standardized acute care surgery (ACS) framework.

**Materials and methods:**

This single center retrospective cohort study analyzed patients diagnosed with GIP who underwent emergent surgery and were admitted to the intensive care unit between January 2013 and March 2023.

**Results:**

Among 354 patients, the mortality was 11%, and 38% of survivors experienced significant complications (Clavien-Dindo class III or higher). Independent prognostic factors for mortality included initial sequential organ failure assessment (SOFA) scores (at the time of admission or ACS activation), postoperative SOFA (p-SOFA) scores, and postoperative body temperatures. For morbidity, independent predictors were the extent of peritonitis, the open surgery, postoperative albumin levels, and p-SOFA scores. These factors showed significant predictive accuracy for patient outcomes, as evidenced by the area under the receiver operating characteristic curve. The Random Forest model identified p-SOFA scores and postoperative albumin levels as the most significant predictors for both survival and complications, with feature importances of 40.46% and 36.61% for survival, and 39.97% and 37.28% for complications, respectively. Postoperative body temperature also played a moderately important role, contributing 14.63% to mortality and 15.9% to morbidity predictions. Patients with a p-SOFA score ≥ 7, postoperative albumin ≤ 2, and body temperature ≤ 36 °C, as well as those with a p-SOFA score ≥ 10, albumin ≤ 2.9, and body temperature ≤ 36 °C, had a 100% mortality rate. These factors are critical indicators for predicting patient outcomes.

**Conclusion:**

It is crucial to establish a system that ensures rapid preoperative work-up, accurate surgical intervention, and evidence-based postoperative critical care. Implementing such a system and assessing patient outcomes after surgery using the identified factors could provide a more detailed evaluation.

**Supplementary Information:**

The online version contains supplementary material available at 10.1186/s12893-024-02687-7.

## Background

Gastrointestinal perforation (GIP), a critical medical emergency, poses significant challenges due to its high mortality and the complexity of its clinical management. The management of GIP has been a focus of extensive medical research, with studies highlighting various prognostic factors that influence patient outcomes. These factors include the site of perforation, the patient’s physiological response, and the timeliness of medical intervention [1–4]. However, upon reviewing existing studies, we found that most were retrospective involving small cohorts. Additionally, we discovered the heterogenity of the treatments received by each patient. We considered that the urgency of the GIP and its high mortality rate might make it challenging to conduct prospective studies using protocolized treatment. Therefore, we focused on patients with GIP who received consistent treatment over an extended period to identify key prognostic factors. In line with this, our study aimed to determine how various clinical factors and established scoring systems, as identified in previous research, influence the prognostic outcomes of this patient cohort, who have been under consistent care for the past decade.

## Methods

This single center retrospective study was approved by the Institutional Review Board of our institution (HC23RISI0121), and has been registered with the Clinical Research Information Service (CRIS, cris.nih.go.kr), which is an agency of our government (KCT0009348). It was described in accordance with the STROCSS criteria [5]. This study included patients treated from January 2013 to March 2023, who met al.l of the following criteria: (1) patients aged 18 and older diagnosed with GIP, including perforation of the esophagus, stomach, duodenum, small intestine, colon, rectum, appendix, either preoperatively or during the operation; (2) patients who underwent emergent surgery performed by two surgeons in the ACS department, who was proficient from a learning curve perspective; and (3) patients who were admitted to the intensive care unit (ICU) postoperatively. Patients with peritonitis without clear evidence of perforation (e.g., ischemic enteritis, retroperitoneal abscess, cholangitis, pancreatitis, cholecystitis) were excluded from the study, while patients with perforated appendicitis requiring postoperative critical care were included. The study’s primary outcome was mortality, and the secondary outcome was post-operative complications (morbidity).

Throughout the study period, all patients received surgery and postoperative critical care following the same protocol by a single ACS team, and the medical records were also documented by the ACS department of our institution. Based on these records, we retrospectively analyzed data to identify factors determining mortality and morbidity. Patients who survived and were discharged were included in the survivor group, while those who died during hospitalization or were given a hopeless discharge, which is defined as the transfer of a patient deemed medically irrecoverable, following the cessation of futile life-sustaining treatment, to allow the patient to spend their final moments at home or a preferred facility were categorized into the non-survivor group. Within the survivor group, postoperative complications were graded by the Clavien-Dindo (CD) classification [6], with complications either absent or classified as CD I–II included in the non-complicated (non-com) group, while class III or higher were placed in the complicated (com) group. Although CD I and II are technically considered minor complications, this study, being a retrospective review based on medical records, could not differentiate between them in detail, and thus they were included in the non-com group. Upon patient arrival at the emergency department or immediately after the ACS team’s evaluation for inpatients, the initial sequential organ failure assessment (SOFA) score, referred to as i-SOFA, is determined. This score assesses organ dysfunction at two critical junctures: for emergency department arrivals, it is the first SOFA score recorded; for inpatients, it is the first SOFA score following ACS activation. Additionally, the SOFA score was measured immediately after surgery upon ICU admission, which is referred to as the postoperative SOFA (p-SOFA). The Acute Physiology and Chronic Health Evaluation (APACHE) II score was calculated using the first set of clinical and laboratory data obtained immediately after the patient was transferred to the ICU following surgery. The level of intensive care was categorized according to the guidelines of the Intensive Care Society [7] into levels 1, 2, and 3. The process to surgery was differentiated based on whether the patient directly presented to the emergency department or was an inpatient at the time of the surgical need. The route of admission was classified into three categories: direct admission, transferred from another hospital, or already an inpatient. The “door to operation” time was determined by referencing medical records, calculating the difference between the patient’s admission time and the time of surgery. For inpatients, the duration was measured from the activation of the ACS team to the time of surgery. Additionally, the time from the onset of symptom (Sx) to hospital arrival and ultimately to ACS team activation was also distinguished and investigated. Hypotension was determined if the patient’s systolic blood pressure (SBP) was below 90 mmHg, or the lactate level was greater than 4 mmol/L, or if vasopressor was required. The presence and severity of peritonitis were assessed macroscopically.

### Statistical analysis

Summary statistics are presented as frequencies and percentages for categorical variables, and as means ± standard deviations for continuous variables. The assumption of normality was assessed using the Shapiro-Wilk test. For categorical variables, the chi-square and Fisher’s exact tests were employed, while for continuous variables, the t-test and Wilcoxon test were utilized to validate the differences observed. Subsequently, univariate logistic regression was conducted for factors that showed significant differences, followed by multivariate regression analysis on those significant factors. Upon identifying statistically significant variables through multivariate analysis, the area under the receiver operating characteristic (ROC) curve (AUC) was calculated to evaluate the predictive value using training data (70%) and testing data (30%). A two-sided *P* value of < 0.05 was considered statistically significant. All statistical analyses were performed using the R software package, version 4.2.1. In the Random Forest machine learning model, each decision tree T(x) is constructed using a random subset of the data and features, with the final prediction being derived from a majority vote for classification or an average for regression: $\:\widehat{\text{y}}=\frac{1}{\text{B}}{{\Sigma\:}}_{\text{b}=1}^{\text{B}}\text{T}\text{b}\left(\text{x}\right)\:,$where B is the total number of trees. The trees are grown by maximizing the reduction in Gini Impurity,$\:\:\text{G}={\sum\:}_{\text{i}=1}^{\text{c}}{\text{p}}_{\text{i}}\left(1-{\text{p}}_{\text{i}}\right)$, or Entropy, H(S$\:)=-{\sum\:}_{\text{i}=1}^{\text{C}}{\text{p}}_{\text{i}}\:log2\left({\text{p}}_{\text{i}}\right)$, where pi represents the proportion of elements belonging to class i. Additionally, the Random Forest model estimates prediction accuracy internally through the Out-of-Bag (OOB) error, calculated as the average error across all observations using only the trees where each observation was not included in the bootstrap sample: OOB Error = $\:\frac{1}{\text{N}}{{\Sigma\:}}_{\text{i}=1}^{\text{N}}\text{L}\left(\text{y}\text{i},\widehat{\text{y}}OOB,\:i\right).\:$The dataset was divided into a training set, comprising 70% of the data, and a test set, containing the remaining 30%. This split ensured that the model could be trained on a substantial portion of the data while retaining enough data to independently evaluate its performance. Models were trained separately for each outcome (survival and complications) using the training data. Each model was constructed using the default parameters of the algorithm, allowing the Random Forest to build multiple decision trees and aggregate their predictions. To quantify the contribution of each variable, feature importance was calculated for each model. In the context of Random Forest, feature importance is derived from the reduction in impurity (Gini importance), indicating how much each variable contributes to the model’s ability to accurately classify outcomes. The resulting importance scores were normalized, providing a clear picture of the relative influence of each predictor on the outcomes. Machine learning analyses, including the implementation of the Random Forest algorithm, were conducted using the Python programming language with the scikit-learn library (version 0.24.2).

## Results

During the study, 354 patients were analyzed, with an 11% mortality rate (42 patients) and 38% of survivors (120 out of 312) experiencing CD III or higher complications. Additional file 1 presents differences between non-survivors and survivors. The time from Sx onset to ACS activation was longer for non-survivors (2.33 days vs. 1.22 days, *P* = 0.04). Colorectal perforation found during the operation was more prevalent in non-survivors (52.38% vs. 32.37%, *P* = 0.02), and the peritonitis due to colorectal origin was significantly higher in non-survivors (54.76% vs. 30.77%, *P* = 0.000). Non-survivor exhibited higher i-SOFA scores (4.59 vs. 1.78, *P* = 0.00), lower ratio of arterial oxygen partial pressure to fractional inspired oxygen (PF) (291.85 vs. 342.66, *P* = 0.01), and lower SBP (123.07 mmHg vs. 136.44 mmHg, *P* = 0.00), with a higher incidence of hypotension (52.38% vs. 21.15%, *P* = 0.00). Postoperatively, worsened clinical indicators in non-survivors included elevated APACHE II score (21.09 vs. 10.43, *P* = 0.00) and p-SOFA score (7.14 vs. 2.89, *P* = 0.00), and lower PF ratio (290.38 vs. 354.19, *P* = 0.02). Body temperature (BT) measurements were consistently lower in non-survivors. Additional file 2 compares non-com and com patients. The interval from Sx onset to hospital visit and ACS activation was longer in the com group (1.47 days and 0.79 days, *P* = 0.00; 1.69 days vs. 0.93 days, *P* = 0.00, respectively). The incidence of colorectal perforation identified during surgery was higher in the com group (36.67% vs. 29.69%, *P* = 0.03), although univariate analysis of colorectal perforation alone showed no significant difference. Preoperative physiological and severity indicators showed a PF ratio of 326 in the com group versus 362 in the non-com group (*P* = 0.03), and an i-SOFA score of 3.09 compared to 0.97 (*P* = 0.03). SBP was lower in the com group (108 mmHg vs. 124 mmHg, *P* = 0.00), with a higher incidence of hypotension (41.67% vs. 8.33%, *P* = 0.00). Postoperatively, the com group exhibited a higher APACHE II score (14.57 vs. 7.85, *P* = 0.00), a lower PF ratio (322 vs. 384, *p* = 0.00), and an elevated p-SOFA score (4.8 vs. 1.69, *P* = 0.00). The com group experienced slightly lower BTs immediately after surgery (36.31 °C vs. 36.45 °C, *p* = 0.01) and the highest BTs on the day of surgery were also lower in the com group (37.06 °C vs. 37.28 °C, *P* = 0.01) compared to the non-com group. In a subgroup analysis of 123 colorectal perforation patients, 4 cases of sterile perforation were excluded, leaving 119 in the final group. The non-colorectal perforation group included 235 patients. Door to operation time showed no significant difference between the groups. Mortality was higher in the colorectal perforation group (19.33% vs. 8.09%, *p* = 0.003), as was morbidity (55.46% vs. 40.43%, *p* = 0.010). Using Pearson’s correlation coefficient, a weak and non-significant negative correlation was found between door to operation time and both mortality (*r* = -0.051) and morbidity (*r* = -0.033) in colorectal perforation patients.

**Fig. 1:**
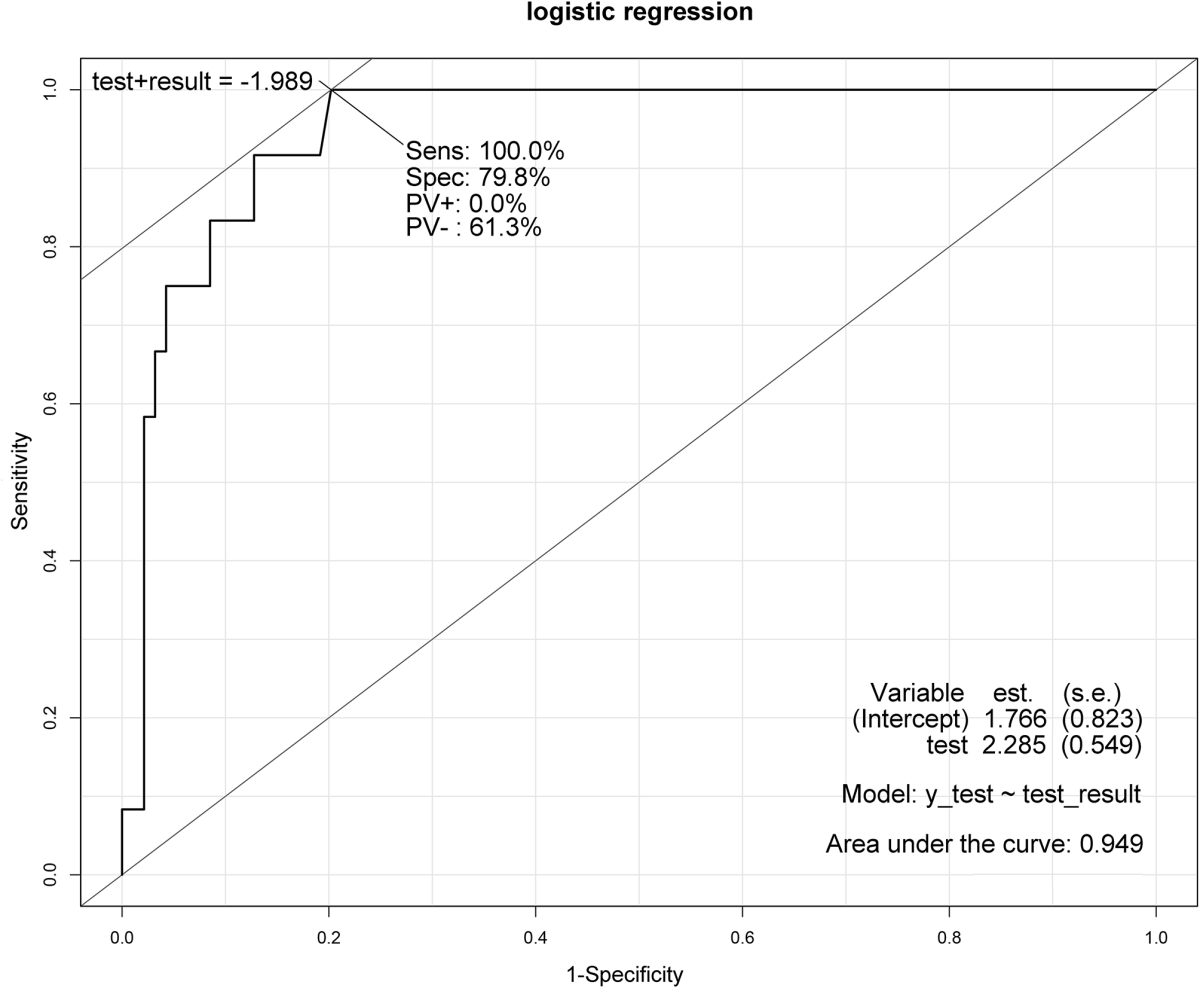
Predictive value of the mortality predictive factors

**Fig. 2:**
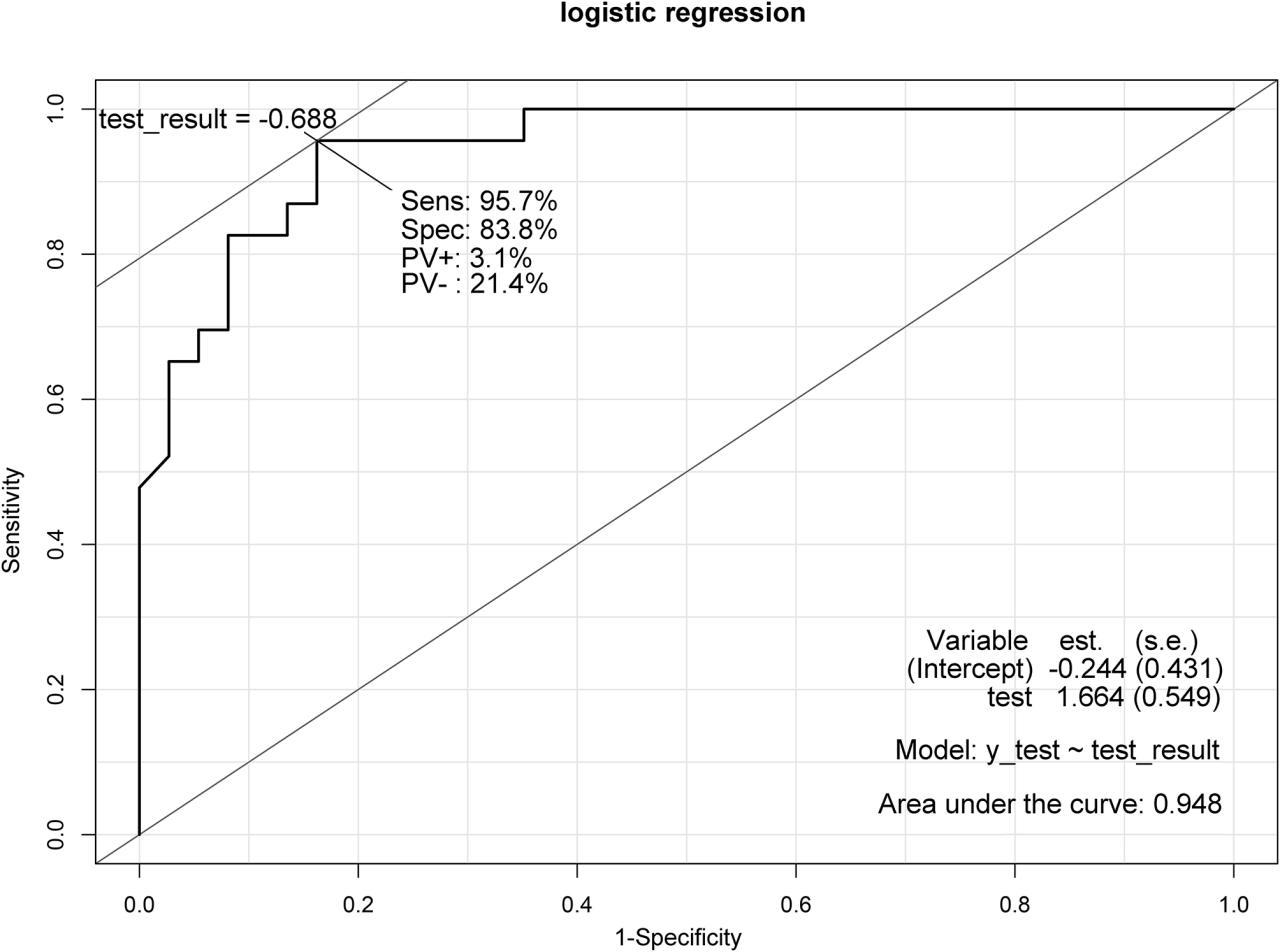
Predictive value of the morbidity predictive factors

After initial comparisons, univariate logistic regression identified significant variables, which were then analyzed via multivariate logistic regression to identify independent risk factors for mortality and morbidity. Outcome-related variables like ICU stay and critical care level were excluded from multivariate analysis for lacking predictive relevance. As a result, i-SOFA and p-SOFA scores exhibited odds ratio (OR)s of 1.44 and 1.22, respectively, delineating them as independent predictive factors for mortality (Table 1). Moreover, the immediate postoperative BT, with an OR of 0.36, indicated that an elevation in BT is inversely associated with mortality risk. The ROC curve, presented in Fig. 1, with an AUC of 0.949, affirmed their substantial predictive accuracy. Table 2 reveals independent predictors for postoperative complications, indicating that patients undergoing open surgery, compared to laparoscopic surgery, experienced a 9.86-fold increase in the likelihood of complications. Furthermore, the risk of complications escalated by 2.56 times with each progression from none to localized to generalized intra-peritoneal contamination. The p-SOFA score showed a positive correlation with an OR of 1.82, while the immediate postoperative albumin level demonstrated an inverse relationship, with an OR of 0.22, suggesting that lower albumin levels significantly increase the risk of complications. The ROC curve for these variables is depicted in Fig. 2, with an AUC of 0.948, confirming their reliable predictive value.

**Table 1:** Independent predictive factors for mortality

Variables	OR	CI (95%)	*P*
i-SOFA	1.44	(1.10, 1.88)	0.01
p-SOFA	1.24	(1.00, 1.54)	0.05
Body temperature, immediate postoperative	0.36	(0.13, 0.37)	0.05

SOFA = sequential organ failure assessment; CI = confidence interval

**Table 2:** Independent predictive factors for postoperative complications

Variables	OR	CI (95%)	*P*
Type of surgery (open vs. laparoscopy)	9.86	(4.24, 22.93)	0.01
Extent of the intra-peritoneal contamination	2.56	(1.71, 3.84)	0.00
p-SOFA	1.82	(1.53, 2.17)	0.00
Serum albumin level, immediate postoperative	0.22	(0.13, 0.37)	0.02

SOFA = sequential organ failure assessment; CI = confidence interval

Based on the identified variables, a machine learning approach was used to evaluate each predictor’s contribution to survival and complications. Open surgery was excluded from the analysis as its associated morbidity is likely due to underlying clinical conditions, such as hemodynamic instability and severe peritonitis, rather than the surgery itself. Variables from traditional methods were combined for a comprehensive assessment of their impact on both mortality and morbidity. For survival (Table 3), the model showed good predictive performance with an accuracy of 88.79% and an ROC-AUC of 0.76. p-SOFA (40.46%) and postoperative albumin level (36.61%) were the most influential predictors, followed by postoperative BT (14.63%). Extent of peritonitis (6.86%) and i-SOFA (1.44%) were less impactful. In the complications analysis (Table 4), the model had an accuracy of 78.50% and an ROC-AUC of 0.84. p-SOFA (39.97%) and postoperative albumin level (37.28%) were again the most important variables, with postoperative BT (15.90%) also contributing significantly. Extent of peritonitis (6.46%) and i-SOFA (0.40%) had minimal impact. The model performed well in predicting both survival and complications, with high precision, recall, and F1-scores.

**Table 3:** Variable importance and performance metrics for predicting mortality using the Random Forest Model

Metric	*p*-SOFA	*p*-alb	*p*-BT	Extent of peritoneal contamination	i-SOFA
Importance (%)	40.46	36.61	14.63	6.86	1.44
Accuracy (%)	88.79	N/A	N/A	N/A	N/A
ROC-AUC	0.76	N/A	N/A	N/A	N/A
Precision (Class 0)	0.93	N/A	N/A	N/A	N/A
Precision (Class 1)	0.55	N/A	N/A	N/A	N/A
Recall (Class 0)	0.95	N/A	N/A	N/A	N/A
Recall (Class 1)	0.46	N/A	N/A	N/A	N/A
F1-score (Class 0)	0.94	N/A	N/A	N/A	N/A
F1-score (Class 1)	0.5	N/A	N/A	N/A	N/A

SOFA = sequential organ failure assessment; p-alb = postoperative serum albumin levels; p-BT = postoperative body temperature

**Table 4:** Variable importance and performance metrics for predicting morbidity using the Random Forest Model

Metric	*p*-SOFA	*p*-alb	*p*-BT	Extent of peritoneal contamination	i-SOFA
Importance (%)	39.97	37.28	15.9	6.46	0.4
Accuracy (%)	78.5	N/A	N/A	N/A	N/A
ROC-AUC	0.84	N/A	N/A	N/A	N/A
Precision (Class 0)	0.87	N/A	N/A	N/A	N/A
Precision (Class 1)	0.68	N/A	N/A	N/A	N/A
Recall (Class 0)	0.9	N/A	N/A	N/A	N/A
Recall (Class 1)	0.72	N/A	N/A	N/A	N/A
F1-score (Class 0)	0.88	N/A	N/A	N/A	N/A
F1-score (Class 1)	0.7	N/A	N/A	N/A	N/A

SOFA = sequential organ failure assessment; p-alb = postoperative serum albumin levels; p-BT = postoperative body temperature

## Discussion

This study identified i-SOFA, p-SOFA and postoperative BT as independent predictors of mortality in patients with GIP. Furthermore, it was determined that undergoing open surgery compared to laparoscopic surgery, the extent of peritonitis, p-SOFA, and postoperative albumin levels serve as independent predictors for complications. However, we suggest that the open surgery, in itself, cannot be the cause of increased morbidity. Instead, it seems the various clinical reasons for opting for open surgery over laparoscopic surgery—namely, hemodynamic instability and the preoperatively assessed severity of peritonitis—that allow for the prediction of higher morbidity rates. Upon further validation using machine learning methods, p-SOFA and postoperative albumin level were identified as the strongest predictors of both mortality and morbidity. Postoperative BT showed moderate importance (14.63% for mortality, 15.9% for morbidity). The Random Forest algorithm was chosen for this study due to its strong performance with complex, high-dimensional clinical data [8]. It effectively handles numerous variables and non-linear interactions, making it ideal for analyzing the multiple clinical factors influencing patient outcomes [9]. Random Forest’s ability to capture these intricate relationships is crucial for accurately assessing predictors of survival and complications. Additionally, its robustness to noise and capacity to generalize well to new data reduces overfitting, enhancing prediction reliability [10]. The model’s interpretability, through clear feature importance measures, is essential for identifying significant clinical predictors and guiding future research [11]. Moreover, its strong performance with imbalanced datasets, such as those with less frequent outcomes like mortality, was particularly advantageous in this study [12].

Based on our study findings, we suggest that the absence of a febrile response in patients with GIP may be an indicator of poor prognosis. Additionally, when combined with the SOFA score, which is widely used in diagnosing and assessing the severity of sepsis, and postoperative albumin levels, this comprehensive approach could further enhance the prediction of outcomes in GIP patients. More specifically, our analysis revealed certain conditions under which the mortality rate was 100%. In the first set, all patients with a p-SOFA score of 7 or higher, a postoperative albumin level of 2 or lower, and a body temperature of 36 °C or lower succumbed. Similarly, in the second set, all patients with a p-SOFA score of 10 or higher, a postoperative albumin level of 2.9 or lower, and a body temperature of 36 °C or lower also demonstrated a 100% mortality rate. These conditions thus serve as critical predictive indicators of patient outcomes in clinical settings. Fever is often a sign of systemic infection or surgical complications, and producing a fever requires a significant metabolic effort, with an estimated 11–13% increase in oxygen consumption for every 1 °C rise in body temperature [13]. This metabolic demand can cause concern among surgeons, prompting them to investigate potential infectious sources. Historically, Cuthbertson, in 1942, categorized the body’s metabolic response to injury into two distinct phases: the ebb and flow phases [14]. During the flow phase, fever is seen as a physiological outcome of an elevated metabolic rate, indicative of the body’s recovery process. While fever is a common occurrence post-operatively, its precise pathophysiological role is still not fully understood. For critically ill patients, extremely high fevers (≥ 39.5 °C) have been linked to higher mortality rates [15], yet in cases of infection, moderate fever may serve as an adaptive response, with higher peak BT in ICU patients correlating with better survival outcomes [16], and low BT has been reported to increase mortality in trauma patients [17]. This demonstrates the dual nature of fever, where it can either signal danger or be part of a beneficial immune response, depending on the context. Postoperative hypoalbuminemia is another crucial predictor of complications. A significant drop in serum albumin after surgery is linked to severe complications, including sepsis, wound infections, and mortality. A drop greater than 15–20% in albumin levels within the first 48 h after surgery can be strongly associated with worse outcomes, reflecting the body’s inflammatory response and surgical stress [18, 19].

Our analysis did not corroborate what had almost been accepted as dogma in previous research. A study involving 35,311 patients at a trauma center [4] identified delayed treatment and incorrect diagnosis as leading causes of preventable death. In light of this, we meticulously analyzed patient timelines, including door to operation, Sx onset to visit, and Sx onset to ACS activation. Although univariate analysis revealed some differences, multivariate analysis did not identify these three variables as predictors of mortality or morbidities. In addition, while it is commonly understood in clinical practice that perforations of the colon and rectum may have a higher likelihood of leading to sepsis compared to GIP with other etiologies, and some studies have reported poorer outcomes for colonic perforation [20, 21], our research did not corroborate these findings. In the subgroup analysis, while higher mortality and morbidity rates were observed in the colorectal perforation group, these findings were not confirmed in the multivariable analysis that accounted for the correlations between various factors. This apparent contradiction across two aspects is presumed to be interconnected. Namely, the treatment of surgical emergencies by the ACS likely experienced lower instances of treatment delays and diagnostic errors compared to conventional settings, and this adherence to a structured care approach is presumed to have led to outcomes that diverge from those reported in previous studies, potentially influencing even cases of colorectal perforation and generalized peritonitis. Although definitive evidence establishing the superiority of outcomes from the ACS model over traditional models for all patients with GIP remains elusive, existing research provides some support for the efficacy of the ACS model. According to a recent systematic review [22], ACS models vary globally due to regional healthcare variations, significantly affecting patient outcomes. Models with dedicated 24/7 ACS teams have shown improved outcomes by minimizing delays in emergency surgeries and reducing complication rates. Regionalized care and hub-and-spoke systems enhance survival and efficiency across varying case severities. While the dedicated ACS model is ideal, resource constraints limit its widespread adoption, with successful implementations mainly in the U.S. and Canada. Our study’s team, which included two surgeons, one intern, and a nurse, operated under a hybrid model due to our inability to exclusively manage all emergency cases.

Our cohort was not sufficiently large to accurately calculate cut-off values due to issues with data distribution, the presence of outlying and missing data, and the resulting complexity and potential unreliability of the model. Additionally, we were unable to directly compare the outcomes of the ACS model and traditional model, and we were also unable to analyze c-reactive protein and procalcitonin levels, which are widely used clinically in patients with sepsis, due to missing data issues. Going forward, well-designed prospective studies applying the ACS model will be necessary to support our research findings. Future research is particularly needed to address confounding bias, specifically standardizing for factors such as patient severity, surgeon’s level, and hospital capacity. It is essential to determine whether there is a difference in patient outcomes between the ACS model and the traditional model after equalizing these factors. Additionally, it is necessary to elucidate the treatment outcomes and prognostic factors when applying the ACS model not only to patients admitted to intensive care units but also to all GIP patients, and furthermore, in all surgical emergencies.

In conclusion, this study established that i-SOFA, p-SOFA, and postoperative BT are independent predictors of mortality in patients with GIP. Additionally, the type of surgery (open versus laparoscopic), the severity of peritonitis, p-SOFA, and postoperative albumin levels were found to independently predict complications. When further validated using machine learning techniques, p-SOFA and postoperative albumin emerged as the most significant predictors for both mortality and morbidity, with postoperative BT also playing a moderately important role. In the context of most surgical emergencies, including GIP, it seems essential to aim for a system that functions reliably, incorporating rapid preoperative work-up, precise surgical intervention, and evidence-based postoperative critical care. The implementation of such a system with the ACS model, followed by an assessment of patient outcomes after surgery using factors identified through our research, could potentially offer a more nuanced evaluation.

## Electronic supplementary material

Below is the link to the electronic supplementary material.

**Supplementary Material 1**: **Additional table 1**: Comparison of differences among the entire patient group, deceased group, and survivor group.

**Supplementary Material 2**: **Additional table 2**: Comparison of differences between the group of survivors who developed complications classified as Clavien-Dindo class III or higher, and those who did not.

## Data Availability

The data of this study is not publicly available, but upon a reasonable request to the corresponding author (jinbum21@catholic.ac.kr), the corresponding author may provide the data after consulting with the other authors if the reason is deemed reasonable.

## Abbreviations

ACS: Acute care surgery
APACHE: Acute physiology and chronic health evaluation
AUC: Area under the curve
BT: Body temperature
CD: Clavien-dindo
Com: Complicated
GIP: Gastrointestinal perforation
ICU: Intensive care unit
i-SOFA: Initial sequential organ failure assessment
Non-com: Noncomplicated
OOB: Out-of-Bag
OR: Odds ratio
p-SOFA: Postoperative sequential organ failure assessment
ROC: Receiver operating characteristic
SBP: Systolic blood pressure
SOFA: Sequential organ failure assessment
Sx: Symptom
