# Supplementary material for: Prognostic factors in patients with gastrointestinal perforation under the acute care surgery model : a retrospective cohort study
Source: BMC Surg. 2024 Dec 21;24:406. doi: 10.1186/s12893-024-02687-7 (PMC11662852; doi:10.1186/s12893-024-02687-7)
Supplement: Supplementary file 1 — Supplementary Material 1: Additional table 1: Comparison of differences among the entire patient group, deceased group, and survivor group. [file 12893_2024_2687_MOESM1_ESM.docx]

**Additional Table 1**

**Comparison of differences among the entire patient group, deceased group, and survivor group.**

|  | **Total (n=354)** | **Survivor (n=312)** | **Nonsurvivor (n=42)** | *P***^*^** |
| --- | --- | --- | --- | --- |
| **Gender** |  |  |  | 0.41 |
| male | 202 (57.06) | 181 (58.01) | 21 (50.00) |  |
| female | 152 (42.94) | 131 (41.99) | 21 (50.00) |  |
| **Age (years)** | 65.89±15.40 | 64.64±15.47 | 75.14±11.13 | 0.00 |
| **Level of intensive care** |  |  |  | 0.00 |
| 1 | 128 (36.16) | 126 (40.38) | 2 (4.76) |  |
| 2 | 142 (40.11) | 133 (42.63) | 9 (21.43) |  |
| 3 | 84 (23.73) | 53 (16.99) | 31 (73.81) |  |
| **ASA physical status** |  |  |  | 0.00 |
| 1 | 46 (12.99) | 46 (14.74) | 0 (0.00) |  |
| 2 | 118 (33.33) | 112 (35.90) | 6 (14.29) |  |
| 3 | 149 (42.09) | 127 (40.71) | 22 (52.38) |  |
| 4 | 36 (10.17) | 24 (7.69) | 12 (28.57) |  |
| 5 | 5 (1.41) | 3 (0.96) | 2 (4.76) |  |
| **Type of surgery** |  |  |  | 0.05 |
| open | 284 (80.23) | 245 (78.53) | 39 (92.86) |  |
| laparoscopy | 70 (19.77) | 67 (21.47) | 3 (7.14) |  |
| **Type of surgery** |  |  |  | 0.00 |
| primary repair or wedge resection | 114 (32.20) | 106 (33.97) | 8 (19.05) |  |
| resection and anastomosis | 130 (36.72) | 115 (36.86) | 15 (35.71) |  |
| resection and diversion | 78 (22.03) | 63 (20.19) | 15 (35.71) |  |
| exploration, definite | 20 (5.65) | 19 (6.09) | 1 (2.38) |  |
| exploration, damage-control | 3 (0.85) | 0 (0.00) | 3 (7.14) |  |
| appendectomy | 9 (2.54) | 9 (2.88) | 0 (0.00) |  |
| **Operation time (mins)** | 143.72± 60.04 | 143.18±59.09 | 147.76±66.50 | 0.88 |
| **Intra-op fluid (ml)** |  |  |  |  |
| crystalloid | 1634.59±1105.71 | 1571.20±1055.86 | 2105.47±1330.97 | 0.01 |
| colloid | 242.43±303.58 | 234.85±294.11 | 307.14±360.05 | 0.12 |
| **Intra-op urine output (ml)** | 220.04±232.05 | 216.19±212.65 | 248.57±341.75 | 0.53 |
| **Door to op (mins)** | 265.90±148.56 | 264.30±147.89 | 278.17±156.83 | 0.59 |
| **Symptom onset to visit (days)** | 1.16±2.95 | 1.05±2.55 | 1.97±4.97 | 0.15 |
| **Symptom onset to ACS activation (days)** | 1.35±3.14 | 1.22±2.74 | 2.33±5.21 | 0.04 |
| **Process to operation** |  |  |  | 0.07 |
| visit to ER | 293 (82.77) | 262 (83.97) | 31 (73.81) |  |
| inpatient (other departments ) | 43 (12.15) | 37 (11.86) | 6 (14.29) |  |
| inpatient (GS) | 18 (5.08) | 13 (4.17) | 5 (11.90) |  |
| **Routes of admission** |  |  |  | 0.12 |
| direct | 318 (89.83) | 281 (90.06) | 37 (88.10) |  |
| transfer from other hospital | 27 (7.63) | 25 (8.01) | 2 (4.76) |  |
| inpatient | 9 (2.54) | 6 (1.92) | 3 (7.14) |  |
| **History of severe organ insufficiency** | 145 (40.96) | 112 (35.09) | 33 (78.57) | 0.00 |
| **Perforated organ on preoperative CT** |  |  |  | 0.38 |
| Stomach | 30 (8.47) | 26 (8.33) | 4 (9.52) |  |
| Duodenum | 48 (13.56) | 46 (14.74) | 2 (4.76) |  |
| Small intestine | 97 (27.40) | 83 (26.60) | 14 (33.33) |  |
| Colorectum | 91 (25.71) | 80 (25.64) | 11 (26.19) |  |
| cannot specify, but can confirm the perforation | 37 (10.45) | 33 (10.58) | 4 (9.52) |  |
| no evidence of perforation | 26 (7.34) | 21 (6.73) | 5 (11.90) |  |
| cannot perform CT scan | 10 (2.82) | 9 (2.88) | 1 (2.38) |  |
| appendix | 12 (3.39) | 12 (3.85) | 0 (0.00) |  |
| esophagus | 3 (0.85) | 2 (0.64) | 1 (2.38) |  |
| **Colorectal perforation on preoperative CT** | 91 (25.71) | 80 (25.64) | 11 (26.19) | 1.00 |
| **Perforated organ on operative findings** |  |  |  | 0.14 |
| stomach | 33 (9.32) | 28 (8.97) | 5 (11.90) |  |
| duodenum | 60 (16.95) | 58 (18.59) | 2 (4.76) |  |
| small intestine | 116 (32.77) | 104 (33.33) | 12 (28.57) |  |
| colorectum | 123 (34.75) | 101 (32.37) | 22 (52.38) |  |
| can not be found | 4 (1.13) | 4 (1.28) | 0 (0.00) |  |
| appendix | 14 (3.95) | 13 (4.17) | 1 (2.38) |  |
| esophagus | 1 (0.28) | 1 (0.32) | 0 (0.00) |  |
| not perforation | 3 (0.85) | 3 (0.96) | 0 (0.00) |  |
| **Colorectal perforation on operative findings** | 123 (34.57) | 101 (32.37) | 22 (52.38) | 0.02 |
| **Cause of the perforation on operative findings** |  |  |  | 0.03 |
| neoplasm, malignancy | 44 (12.43) | 38 (12.18) | 6 (14.29) |  |
| neoplasm, benign | 2 (0.56) | 2 (0.64) | 0 (0.00) |  |
| trauma | 32 (9.04) | 31 (9.94) | 1 (2.38) |  |
| intestinal obstruction | 60 (16.95) | 53 (16.99) | 7 (16.67) |  |
| mesenteric ischemia | 30 (8.47) | 19 (6.09) | 11 (26.19) |  |
| iatrogenic | 27 (7.63) | 24 (7.69) | 3 (7.14) |  |
| foreign body-related | 1 (0.28) | 1 (0.32) | 0 (0.00) |  |
| inflammatory bowel disease | 6 (1.69) | 6 (1.92) | 0 (0.00) |  |
| appendicitis | 15 (4.24) | 14 (4.49) | 1 (2.38) |  |
| peptic ulcer | 78 (22.03) | 73 (23.40) | 5 (11.90) |  |
| diverticular, colo-rectal | 18 (5.08) | 17 (5.45) | 1 (2.38) |  |
| diverticular, non-colonic | 3 (0.85) | 3 (0.96) | 0 (0.00) |  |
| tuberculosis | 4 (1.13) | 3 (0.96) | 1 (2.38) |  |
| can not be specified | 34 (9.60) | 28 (8.97) | 6 (14.29) |  |
| **Final pathology** |  |  |  | 0.54 |
| neoplasm, malignancy | 43 (12.15) | 35 (11.22) | 8 (19.05) |  |
| neoplasm, benign | 6 (1.69) | 6 (1.92) | 0 (0.00) |  |
| inflammation | 119 (33.62) | 103 (33.01) | 16 (38.10) |  |
| ischemia | 19 (5.37) | 15 (4.81) | 4 (9.52) |  |
| inflammatory bowel disease | 4 (1.13) | 4 (1.28) | 0 (0.00) |  |
| appendicitis | 14 (3.95) | 14 (4.49) | 0 (0.00) |  |
| peptic ulcer | 22 (6.21) | 19 (6.09) | 3 (7.14) |  |
| diverticular | 17 (4.80) | 15 (4.81) | 2 (4.76) |  |
| tuberculosis | 1 (0.28) | 1 (0.32) | 0 (0.00) |  |
| do not perform | 109 (30.79) | 100 (32.05) | 9 (21.43) |  |
| **Source of the peritonitis** |  |  |  | 0.12 |
| stomach, duodenum, esophagus | 94 (26.55) | 87 (27.88) | 7 (16.67) |  |
| small intestine | 104 (29.38) | 94 (30.13) | 10 (23.81) |  |
| colorectum | 119 (33.62) | 96 (30.77) | 23 (54.76) |  |
| hepatobiliary | 1 (0.28) | 1 (0.32) | 0 (0.00) |  |
| appendicitis | 16 (4.52) | 15 (4.81) | 1 (2.38) |  |
| none | 19 (5.37) | 18 (5.77) | 1 (2.38) |  |
| retroperitoneum | 1 (0.28) | 1 (0.32) | 0 (0.00) |  |
| **Peritonitis by colorectal origin** | 119 (33.62) | 96 (30.77) | 23 (54.76) | 0.00 |
| **Extent of the peritonitis** |  |  |  | 0.46 |
| generalized | 202 (57.06) | 176 (56.41) | 26 (61.90) |  |
| localized | 116 (32.77) | 102 (32.69) | 14 (33.33) |  |
| none | 36 (10.17) | 34 (10.90) | 2 (4.76) |  |
| **Length of ICU stay (days)** | 5.02 ± 5.17 | 4.68±4.79 | 7.57 ± 6.97 | 0.00 |
| **Post-operative length of hospital stay (days)** | 18.01 ± 13.08 | 18.45±12.57 | 14.76 ± 16.22 | 0.00 |
| **Clinical parameters, estimated at first** |  |  |  |  |
| hypotension | 88 (24.86) | 66 (21.15) | 22 (52.38) | 0.00 |
| PF ratio | 333.39 ± 93.90 | 342.66±87.24 | 291.85 ± 111.95 | 0.01 |
| SOFA score | 2.12 ± 2.61 | 1.78 ±2.27 | 4.59 ± 3.49 | 0.00 |
| systolic Blood pressure (mmHg) | 134.85 ± 27.19 | 136.44 ±26.44 | 123.07 ± 30.02 | 0.00 |
| **Laboratory results, estimated at first** |  |  |  |  |
| white blood cell count (/mL) | 11246.39 ± 5960.99 | 11060±5423.44 | 12660.24 ± 9029.40 | 0.80 |
| hemoglobin (g/dL) | 12.80 ± 2.92 | 12.79 ±2.67 | 12.90 ± 4.35 | 0.30 |
| hematocrit (%) | 37.55 ± 6.90 | 37.76 ±6.96 | 35.99 ± 6.29 | 0.07 |
| platelet (10^9^/L) | 522.59 ± 4830.81 | 267.82 ±121.72 | 240.90 ± 140.37 | 0.76 |
| blood urea nitrogen (mg/dL) | 26.49 ± 38.44 | 25.28 ±39.80 | 35.73 ± 24.16 | 0.00 |
| creatinine (mg/dL) | 1.47 ± 1.78 | 1.38 ±1.74 | 2.16 ± 1.89 | 0.00 |
| AST (U/L) | 36.70 ± 81.99 | 37.01 ±86.93 | 34.40 ± 21.97 | 0.00 |
| ALT (U/L) | 27.64 ± 55.73 | 28.10 ±58.62 | 24.20 ± 25.15 | 0.59 |
| Total bilirubin (mg/dL) | 0.84 ± 0.89 | 0.82 ±0.91 | 0.99 ± 0.75 | 0.01 |
| albumin (g/dL) | 3.73 ± 0.71 | 3.77 ±0.70 | 3.41 ± 0.71 | 0.00 |
| **Clinical parameters, immediate postoperative** |  |  |  |  |
| APACHE II score | 11.70 ± 7.56 | 10.43 ±6.45 | 21.09 ± 8.59 | 0.00 |
| PF ratio | 345.39 ± 133.93 | 354.19 ±132.24 | 290.38 ± 133.13 | 0.02 |
| SOFA score | 3.39 ± 3.22 | 2.89 ±2.73 | 7.14 ± 4.00 | 0.00 |
| **Laboratory results, immediate postoperative** |  |  |  |  |
| white blood cell count (/mL) | 11159.61±7403.75 | 11429.67±7139.14 | 9134.25±8997.52 | 0.00 |
| hemoglobin (g/dL) | 12.08 ± 2.06 | 12.21 ±2.05 | 11.10 ± 1.86 | 0.00 |
| hematocrit (%) | 35.67 ± 5.77 | 36.01 ±5.77 | 33.12 ± 5.16 | 0.00 |
| platelet (10^9^/L) | 392.13 ± 302.69 | 235.21 ±117.71 | 156.90 ± 88.274 | 0.00 |
| blood urea nitrogen (mg/dL) | 23.39 ± 16.32 | 22.11 ±15.12 | 32.97 ± 21.28 | 0.00 |
| creatinine (mg/dL) | 1.29 ± 1.58 | 1.21 ±1.53 | 1.87 ± 1.81 | 0.00 |
| AST (U/L) | 44.24 ± 124.70 | 44.61 ±132.31 | 41.45 ± 31.64 | 0.02 |
| ALT (U/L) | 27.49 ± 52.86 | 28.01 ±55.71 | 23.65 ± 22.26 | 0.79 |
| Total bilirubin (mg/dL) | 0.94 ± 0.73 | 0.92 ±0.70 | 1.07 ± 0.91 | 0.64 |
| albumin (g/dL) | 2.94 ± 0.72 | 3±0.70 | 2.51 ± 0.73 | 0.00 |
| **Body temperature (°C)** |  |  |  |  |
| at the time of admission or ACS activation | 36.89 ± 0.75 | 36.93±0.74 | 36.67 ± 0.76 | 0.05 |
| post-op, immediate | 36.37 ± 0.55 | 36.40 ±0.57 | 36.12 ± 0.36 | 0.00 |
| peak body temperature (operation day) | 37.15 ± 0.75 | 37.2±0.72 | 36.85 ± 0.87 | 0.00 |
| peak body temperature (first postoperative day) | 37.50 ± 0.58 | 37.53 ±0.53 | 37.26 ± 0.86 | 0.01 |
| **RBC transfusion** |  |  |  |  |
| patients who received RBC before surgery | 77 (21.75) | 60 (19.23) | 17 (40.48) | 0.70 |
| amount during the operation (units) | 0.49 ± 1.24 | 0.40 ±1.04 | 1.16 ± 2.16 | 0.00 |
| amount within 24 hrs after surgery (units) | 0.25 ± 0.84 | 0.23 ±0.73 | 0.40 ± 1.43 | 0.26 |
| total amount after surgery (units) | 1.05 ± 2.01 | 0.93 ±1.85 | 1.92 ± 2.81 | 0.00 |
| **Patients received mechanical ventilation after surgery** | 73 (20.62) | 44 (14.10) | 29 (69.05) | 0.00 |
| **Patients received continuous renal replacement therapy after surgery** | 37 (10.45) | 19 (6.09) | 18 (42.86) | 0.00 |
| **Patients who developed delirium after surgery** | 125 (35.31) | 99 (31.73) | 26 (61.90) | 0.00 |

Values are presented as number (%), or mean±standard deviation (range).

^*^Survivor vs. Nonsurvivor

ASA = American society of anesthesiologists; op = operation; ACS = acute care surgery; ER = emergency room; GS = general surgery; CT = computed tomography; ICU = intensive care unit; PF = ratio of arterial oxygen partial pressure to fractional inspired oxygen; SOFA = sequential organ failure assessment; AST = aspartate transaminase; ALT = alanine transaminase; APACHE = acute physiology and chronic health evaluation; RBC = red blood cell
