# Supplementary material for: Prognostic factors in patients with gastrointestinal perforation under the acute care surgery model : a retrospective cohort study
Source: BMC Surg. 2024 Dec 21;24:406. doi: 10.1186/s12893-024-02687-7 (PMC11662852; doi:10.1186/s12893-024-02687-7)
Supplement: Supplementary file 2 — Supplementary Material 2: Additional table 2: Comparison of differences between the group of survivors who developed complications classified as Clavien-Dindo class III or higher, and those who did not. [file 12893_2024_2687_MOESM2_ESM.docx]

**Additional Table 2**

**Comparison of differences between the group of survivors who developed complications classified as Clavien-Dindo class III or higher, and those who did not.**

|  | **Non-complicated (n=192)** | **Complicated (n=120)** | ***P*** |
| --- | --- | --- | --- |
| **Gender** |  | m | 0.98 |
| male | 112 (58.33) | 69 (57.50) |  |
| female | 80 (41.67) | 51 (42.50) |  |
| **Age (years)** | 61.32 ± 16.38 | 69.95 ± 12.29 | 0.00 |
| **Level of intensive care** |  |  | 0.00 |
| 1 | 117 (60.94) | 9 (7.50) |  |
| 2 | 71 (36.98) | 62 (51.67) |  |
| 3 | 4 (2.08) | 49 (40.83) |  |
| **ASA physical status** |  |  | 0.00 |
| 1 | 38 (19.79) | 8 (6.67) |  |
| 2 | 86 (44.79) | 26 (21.67) |  |
| 3 | 63 (32.81) | 64 (53.33) |  |
| 4 | 5 (2.60) | 19 (15.83) |  |
| 5 | 0 (0.00) | 3 (2.50) |  |
| **Type of surgery** |  |  | 0.00 |
| open | 132 (68.75) | 113 (94.17) |  |
| laparoscopy | 60 (31.25) | 7 (5.83) |  |
| **Type of surgery** |  |  | 0.00 |
| primary repair or wedge resection | 81 (42.19) | 25 (20.83) |  |
| resection and anastomosis | 67 (34.90) | 48 (40.00) |  |
| resection and diversion | 25 (13.02) | 38 (31.67) |  |
| exploration, definite | 15 (7.81) | 4 (3.33) |  |
| exploration, damage-control | 0 (0.00) | 0 (0.00) |  |
| appendectomy | 4 (2.08) | 5 (4.17) |  |
| **Operation time (mins)** | 136.00 ± 60.78 | 154.67 ± 54.85 | 0.00 |
| **Intra-op fluid (ml)** |  |  |  |
| crystalloid | 1293.56 ± 905.62 | 2015.41 ± 1132.78 | 0.00 |
| colloid | 193.07 ± 266.33 | 301.70 ± 324.98 | 0.00 |
| **Intra-op urine output (ml)** | 204.67 ± 199.75 | 234.53 ± 232.22 | 0.19 |
| **Door to op (mins)** | 264.07 ± 154.22 | 264.67 ± 137.79 | 0.61 |
| **Symptom onset to visit (days)** | 0.79 ± 1.96 | 1.47 ± 3.25 | 0.00 |
| **Symptom onset to ACS activation (days)** | 0.93 ± 2.21 | 1.69 ± 3.38 | 0.00 |
| **Process to operation** |  |  |  |
| visit to ER | 165 (85.94) | 97 (80.83) |  |
| inpatient ( other departments ) | 23 (11.98) | 14 (11.67) |  |
| inpatient (GS) | 4 (2.08) | 9 (7.50) |  |
| **Routes of admission** |  |  | 0.05 |
| direct | 179 (93.23) | 102 (85.00) |  |
| transfer from other hospital | 11 (5.73) | 14 (11.67) |  |
| inpatient | 2 (1.04) | 4 (3.33) |  |
| **History of severe organ insufficiency** | 47 (24.48) | 65 (54.17) | 0.00 |
| **Perforated organ on preoperative CT** |  |  | 0.17 |
| Stomach | 14 (7.29) | 12 (10.00) |  |
| Duodenum | 35 (18.23) | 11 (9.17) |  |
| Small intestine | 54 (28.13) | 28 (23.33) |  |
| Colorectum | 45 (23.44) | 36 (30.00) |  |
| cannot specify, but can confirm the perforation | 19 (9.90) | 14 (11.67) |  |
| no evidence of perforation | 12 (6.25) | 9 (7.50) |  |
| cannot perform CT scan | 7 (3.65) | 2 (1.67) |  |
| appendix | 5 (2.60) | 7 (5.83) |  |
| esophagus | 1 (0.52) | 1 (0.83) |  |
| **Colorectal perforation on preoperative CT** | 45 (23.44) | 36 (30.00) | 0.21 |
| **Perforated organ on operative findings** |  |  | 0.03 |
| stomach | 18 (9.38) | 10 (8.33) |  |
| duodenum | 45 (23.44) | 13 (10.83) |  |
| small intestine | 60 (31.25) | 44 (36.67) |  |
| colorectum | 57 (29.69) | 44 (36.67) |  |
| can not be found | 4 (2.08) | 0 (0.00) |  |
| appendix | 7 (3.65) | 6 (5.00) |  |
| esophagus | 0 (0.00) | 1 (0.83) |  |
| not perforation | 1 (0.52) | 2 (1.67) |  |
| **Colorectal perforation on operative findings** | 57 (29.69) | 44 (36.67) | 0.25 |
| **Cause of the perforation on operative findings** |  |  | 0.00 |
| neoplasm, malignancy | 22 (11.46) | 16 (13.33) |  |
| neoplasm, benign | 2 (1.04) | 0 (0.00) |  |
| trauma | 20 (10.42) | 11 (9.17) |  |
| intestinal obstruction | 31 (16.15) | 22 (18.33) |  |
| mesenteric ischemia | 5 (2.60) | 14 (11.67) |  |
| iatrogenic | 17 (8.85) | 7 (5.83) |  |
| foreign body-related | 1 (0.52) | 0 (0.00) |  |
| inflammatory bowel disease | 6 (3.13) | 0 (0.00) |  |
| appendicitis | 6 (3.13) | 8 (6.67) |  |
| peptic ulcer | 55 (28.65) | 18 (15.00) |  |
| diverticular, colo-rectal | 13 (6.77) | 4 (3.33) |  |
| diverticular, non-colonic | 2 (1.04) | 1 (0.83) |  |
| tuberculosis | 0 (0.00) | 3 (2.50) |  |
| can not be specified | 12 (6.25) | 16 (13.33) |  |
| **Final pathology** |  |  | 0.00 |
| neoplasm, malignancy | 19 (9.90) | 16 (13.33) |  |
| neoplasm, benign | 4 (2.08) | 2 (1.67) |  |
| inflammation | 51 (26.56) | 52 (43.33) |  |
| ischemia | 6 (3.13) | 9 (7.50) |  |
| inflammatory bowel disease | 3 (1.56) | 1 (0.83) |  |
| appendicitis | 6 (3.13) | 8 (6.67) |  |
| peptic ulcer | 11 (5.73) | 8 (6.67) |  |
| diverticular | 12 (6.25) | 3 (2.50) |  |
| tuberculosis | 0 (0.00) | 1 (0.83) |  |
| do not perform | 80 (41.67) | 20 (16.67) |  |
| **Source of the peritonitis** |  |  | 0.00 |
| stomach, duodenum, esophagus | 63 (32.81) | 24 (20.00) |  |
| small intestine | 49 (25.52) | 45 (37.50) |  |
| colorectum | 53 (27.60) | 43 (35.83) |  |
| hepatobiliary | 1 (0.52) | 0 (0.00) |  |
| appendicitis | 7 (3.65) | 8 (6.67) |  |
| none | 18 (9.38) | 0 (0.00) |  |
| retroperitoneum | 1 (0.52) | 0 (0.00) |  |
| **Peritonitis by colorectal origin** | 53 (27.60) | 43 (35.83) | 0.16 |
| **Extent of the peritonitis** |  |  | 0.00 |
| generalized | 95 (49.48) | 81 (67.50) |  |
| localized | 67 (34.90) | 35 (29.17) |  |
| none | 30 (15.63) | 4 (3.33) |  |
| **Length of ICU stay (days)** | 2.82 ± 1.11 | 7.65 ± 6.59 | 0.00 |
| **Post-operative length of hospital stay (days)** | 12.73 ± 4.91 | 27.61 ± 15.38 | 0.00 |
| **Clinical parameters, estimated at first** |  |  |  |
| hypotension | 16 (8.33) | 50 (41.67) | 0.00 |
| PF ratio | 362.01 ± 74.38 | 326.53 ± 94.20 | 0.03 |
| SOFA score | 0.97 ± 1.31 | 3.09 ± 2.83 | 0.00 |
| systolic Blood pressure (mmHg) | 124.20 ± 21.33 | 108.36 ± 26.59 | 0.00 |
| **Laboratory results, estimated at first** |  |  |  |
| white blood cell count (/mL) | 11151.72 ± 4796.46 | 10914.00 ± 6313.21 | 0.35 |
| hemoglobin (g/dL) | 13.17 ± 2.41 | 12.18 ± 2.96 | 0.00 |
| hematocrit (%) | 38.72 ± 6.20 | 36.23 ± 7.81 | 0.00 |
| platelet (10^9^/L) | 262.48 ± 113.04 | 276.33 ± 134.45 | 0.52 |
| blood urea nitrogen (mg/dL) | 20.25 ± 14.03 | 33.34 ± 61.00 | 0.00 |
| creatinine (mg/dL) | 1.26 ± 2.00 | 1.57 ± 1.20 | 0.00 |
| AST (U/L) | 30.81 ± 49.84 | 47.28 ± 126.01 | 0.85 |
| ALT (U/L) | 27.54 ± 53.64 | 29.03 ± 66.29 | 0.15 |
| Total bilirubin (mg/dL) | 0.81 ± 0.95 | 0.83 ± 0.83 | 0.55 |
| albumin (g/dL) | 3.99 ± 0.61 | 3.42 ± 0.69 | 0.00 |
| **Clinical parameters, immediate postoperative** |  |  |  |
| APACHE II score | 7.85 ± 4.19 | 14.57 ± 7.24 | 0.00 |
| PF ratio | 384.11 ± 131.63 | 322.35 ± 125.84 | 0.00 |
| SOFA score | 1.69 ± 1.44 | 4.80 ± 3.20 | 0.00 |
| **Laboratory results, immediate postoperative** |  |  |  |
| white blood cell count (/mL) | 12679.72 ± 6958.71 | 9528.31 ± 7016.89 | 0.00 |
| hemoglobin (g/dL) | 12.59 ± 1.98 | 11.64 ± 2.04 | 0.00 |
| hematocrit (%) | 36.92 ± 5.78 | 34.63 ± 5.50 | 0.00 |
| platelet (10^9^/L) | 244.22 ± 119.07 | 221.51 ± 114.75 | 0.02 |
| blood urea nitrogen (mg/dL) | 19.04 ± 13.35 | 26.81 ± 16.48 | 0.00 |
| creatinine (mg/dL) | 1.12 ± 1.78 | 1.35 ± 1.02 | 0.00 |
| AST (U/L) | 35.00 ± 43.41 | 59.36 ± 203.29 | 0.52 |
| ALT (U/L) | 27.27 ± 42.87 | 29.12 ± 71.10 | 0.01 |
| Total bilirubin (mg/dL) | 0.93 ± 0.68 | 0.91 ± 0.74 | 0.80 |
| albumin (g/dL) | 3.27 ± 0.62 | 2.57 ± 0.59 | 0.00 |
| **Body temperature (°C)** |  |  |  |
| at the time of admission or ACS activation | 36.90 ± 0.74 | 36.97 ± 0.74 | 0.37 |
| post-op, immediate | 36.45 ± 0.59 | 36.31 ± 0.51 | 0.01 |
| peak body temperature (operation day) | 37.28 ± 0.64 | 37.06 ± 0.82 | 0.01 |
| peak body temperature (first postoperative day) | 37.56 ± 0.47 | 37.48 ± 0.61 | 0.27 |
| **RBC transfusion** |  |  |  |
| patients who received RBC before surgery | 4 (2.08) | 11 (9.17) | 0.01 |
| amount during the operation (units) | 0.17 ± 0.65 | 0.75 ± 1.39 | 0.00 |
| amount within 24 hrs after surgery (units) | 0.11 ± 0.48 | 0.41 ± 0.99 | 0.00 |
| total amount after surgery (units) | 0.33 ± 0.93 | 1.89 ± 2.45 | 0.00 |
| **Patients received mechanical ventilation after surgery** | 2 (1.04) | 42 (35.00) | 0.00 |
| **Patients received continuous renal replacement therapy after surgery** | 4 (2.08) | 15 (12.50) | 0.00 |
| **Patients who developed delirium after surgery** | 25 (13.02) | 74 (61.67) | 0.00 |

Values are presented as number (%), or mean±standard deviation (range).

ASA = American society of anesthesiologists; op = operation; ACS = acute care surgery; ER = emergency room; GS = general surgery; CT = computed tomography; ICU = intensive care unit; PF = ratio of arterial oxygen partial pressure to fractional inspired oxygen; SOFA = sequential organ failure assessment; AST = aspartate transaminase; ALT = alanine transaminase; APACHE = acute physiology and chronic health evaluation; RBC = red blood cell
